# Rice intermediate filament, OsIF, stabilizes photosynthetic machinery and yield under salinity and heat stress

**DOI:** 10.1038/s41598-018-22131-0

**Published:** 2018-03-06

**Authors:** Neelam Soda, Brijesh K. Gupta, Khalid Anwar, Ashutosh Sharan, Govindjee, Sneh L. Singla-Pareek, Ashwani Pareek

**Affiliations:** 1 Stress Physiology and Molecular Biology Laboratory, School of Life Sciences, Jawaharlal Nehru University, New Delhi, 110067 India; 2 Plant Stress Biology, International Centre for Genetic Engineering and Biotechnology, Aruna Asaf Ali Marg, New Delhi, 110067 India; 3 Department of Biochemistry, Center of Biophysics & Quantitative Biology, University of Illinois at Urbana-Champaign, 265 Morrill Hall, 505 South Goodwin Av, Urbana, IL 61801-3707 USA; 4 The UWA Institute of Agriculture, School of Agriculture and Environment, The University of Western Australia, Perth, WA Australia

**Keywords:** Gene expression profiling, Abiotic

## Abstract

Cytoskeleton plays a vital role in stress tolerance; however, involvement of intermediate filaments (IFs) in such a response remains elusive in crop plants. This study provides clear evidence about the unique involvement of IFs in cellular protection against abiotic stress in rice. Transcript abundance of *Oryza sativa* intermediate filament (*OsIF)* encoding gene showed 2–10 fold up-regulation under different abiotic stress. Overexpression of *OsIF* in transgenic rice enhanced tolerance to salinity and heat stress, while its knock-down (KD) rendered plants more sensitive thereby indicating the role of IFs in promoting survival under stress. Seeds of OsIF overexpression rice germinated normally in the presence of high salt, showed better growth, maintained chloroplast ultrastructure and favourable K^+^/Na^+^ ratio than the wild type (WT) and KD plants. Analysis of photosynthesis and chlorophyll *a* fluorescence data suggested better performance of both photosystem I and II in the OsIF overexpression rice under salinity stress as compared to the WT and KD. Under salinity and high temperature stress, OsIF overexpressing plants could maintain significantly high yield, while the WT and KD plants could not. Further, metabolite profiling revealed a 2–4 fold higher accumulation of proline and trehalose in OsIF overexpressing rice than WT, under salinity stress.

## Introduction

Plants are often exposed to several unfavourable conditions, such as very low (or very high) temperature, salinity, drought, flooding, oxidative stress and heavy metal toxicity; all of these impose harmful effects on plants leading to reduced yield and productivity^1^. Abiotic stress decreases both growth and productivity of plants by interfering with cellular homeostasis, and reducing photosynthesis^2,3^. Salinity is a major problem in many rice fields because of the sensitivity of most rice cultivars to high salt. Thus, engineering salt tolerant high yielding rice genotypes is of importance for the future. In response to many types of stress, various plant genes undergo differential expression, which can mitigate their effect and lead to an adjustment of the cellular milieu. In rice, several salt stress related Quantitative Trait Loci (QTL) have been reported, which contribute to stress tolerance. *Saltol* is one of the major QTL present on chromosome 1 of rice and is responsible for more than 40% of salinity tolerance at the seedling stage^4^. Our research group has reported^5^ differential expression of *Saltol* QTL related intermediate filaments (IFs) encoding gene, *OsIF*, in salt-tolerant Pokkali and salt-sensitive IR64 rice. *OsIF* remained constitutively expressed at a certain level in Pokkali rice, while in IR64 rice, its expression was induced in response to salinity.

Furthermore, ectopic expression of OsIF in a range of diverse organisms from bacteria to yeast to the model plant tobacco has been reported to improve their survival under stress, suggesting involvement of intermediate filaments in abiotic stress tolerance^6^. To better understand the link between abiotic stress tolerance and intermediate filaments, we raised rice (*Oryza sativa* L. cv. IR64) transgenic plants with overexpression or knockdown of *OsIF* gene. Our results, presented in this paper, clearly demonstrate that overexpression of the *OsIF* gene improves growth of rice transgenic plants under abiotic stress by stabilizing its photosynthesis, which is partly due to unchanged ultrastructure of chloroplasts, which in turn, increases survival and yield of plants. This improved salinity stress tolerance of overexpressed lines is also linked with increased levels of compatible osmolytes such as proline and trehalose in these plants.

## Results

### Multiple alignment and phylogenetic analysis of OsIF

Database search with OsIF as query, gave us only two proteins, one from *Zea mays* (ZmIF) sharing 57% identity and the other from Arabidopsis (AtIF) showing 32% identity with OsIF (Supplementary Fig. S1A). Dendrogram drawn from the amino acid sequence also shows that the OsIF protein (LOC_Os01g18840) is phylogenetically closer to ZmIF than AtIF (Supplementary Fig. S1B).

### *OsIF* expression is induced by abiotic stress

**Figure 1:**
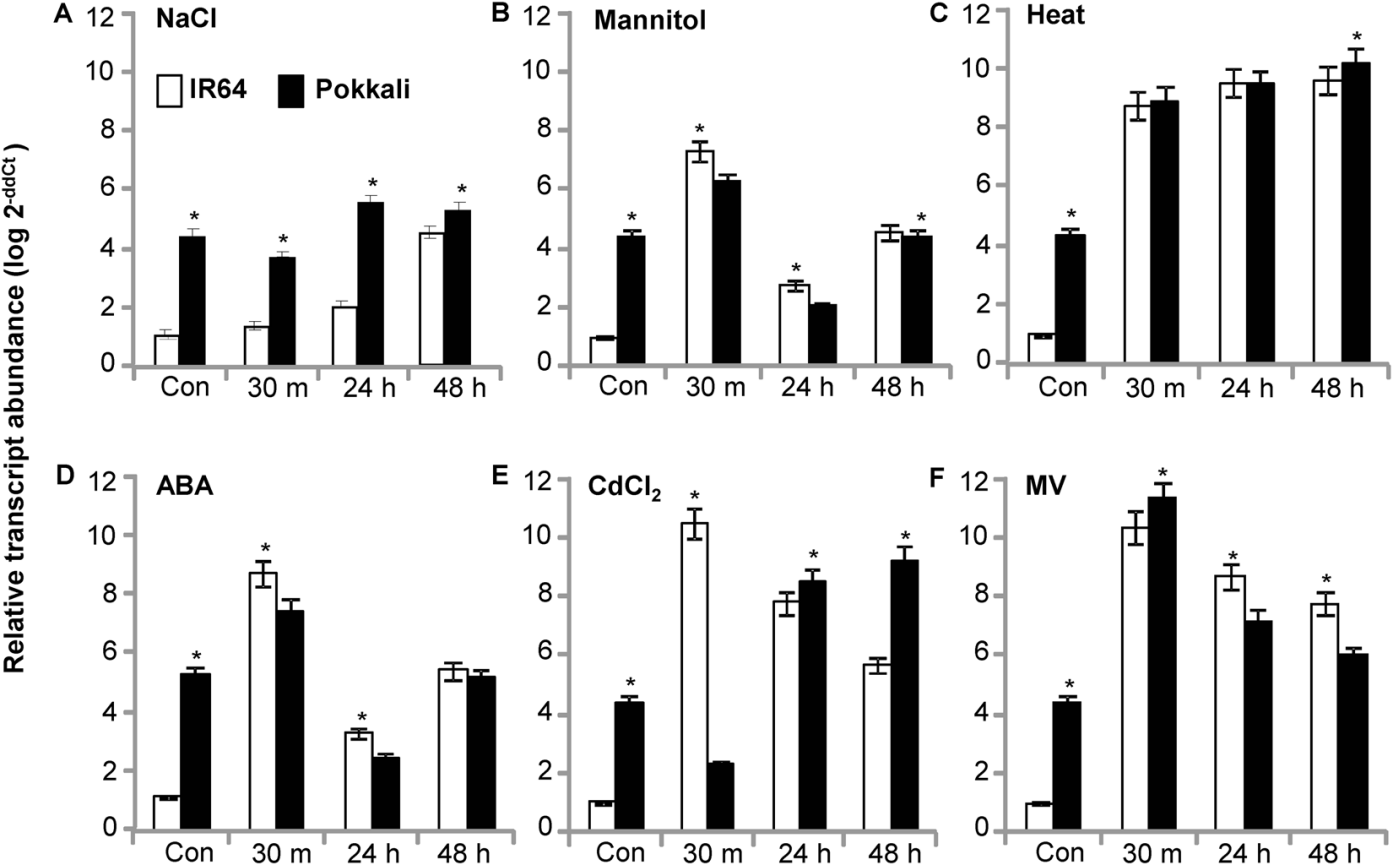
*OsIF* gene is differentially expressed in seedlings of contrasting rice genotypes (IR64 and Pokkali) under various abiotic stresses. Transcript abundance of *OsIF* gene in 7 day old seedlings under control condition and in response to various abiotic stress such as (**A**) 0.2 M NaCl; (**B**) 0.5 M Mannitol; (**C**) 42 °C Heat; (**D**) 100 µM Abscissic acid (ABA); (**E**) 10 µM CdCl_2_; (**F**) 1 µM Methyl viologen (MV). Relative expression of OsIF is represented on the Y-axis as log 2^-ddCt^ (base 10) in the presence of different stressors. Expression values of IR64 control is taken as a reference in each case for the calculation of ddCt values. Con: control (non-stress); 30 m: 30 min stress; 24 h: 24 h stress; 48 h: 48 h stress.

RNA transcript abundance analysis showed a relatively higher (~4 fold) constitutive expression of *OsIF* in Pokkali than in IR64 under control condition (Fig. 1A–F). However, after 48 h of salt stress, there was a 4-fold induction in its transcript abundance in IR64 as compared to control seedlings (Fig. 1A). Under all stress durations, the transcript abundance of *OsIF* was always higher in Pokkali than in IR64. In the presence of osmotic stress (0.5 M mannitol), *OsIF* transcript increased ~7 fold in the IR64 during the early stages of stress (Fig. 1B). Pokkali showed first an increase in the transcript abundance, which then decreased at 24 h of stress. With stress of longer duration, both the genotypes maintained almost equal level of transcripts, which was similar to the initial transcript level of *OsIF* in Pokkali under non-stress conditions (Fig. 1B). The above observations suggest that the higher transcript level of *OsIF* in Pokkali than in IR64, under non-stress condition, may be a contributing factor for its tolerance towards abiotic stress.

High temperature (42 °C) led to 8–9 fold increase in the transcript abundance of *OsIF* gene in both Pokkali and IR64, suggesting an involvement of this gene in heat stress tolerance (Fig. 1C). In response to exogenous abscisic acid, ABA (100 µM), the transcript abundance of *OsIF* in IR64 and Pokkali seedlings showed a pattern which was similar to that under osmotic stress (Fig. 1D). In the presence of heavy metal (such as 10 µM CdCl_2_), IR64 showed a large (10 fold) increase in transcript abundance within 30 min of treatment and then a decrease at 24 h and at 48 h, whereas, Pokkali showed a gradual increase in transcript abundance during the same time period. At 48 h of stress, Pokkali maintained a higher expression of *OsIF* than IR64 (Fig. 1E). Oxidative stress (imposed by the addition of 1 µM methylviologen) led to an increased expression of *OsIF* gene in both the genotypes (6–10 fold) followed by a gradual decrease with time, but it was maintained at a higher level than under non-stress conditions (Fig. 1F). These results suggest that OsIF is a key player in tolerance towards multiple abiotic stresses in rice at the seedling stage.

### Salinity stress induces redistribution of OsIF within the cell

**Figure 2:**
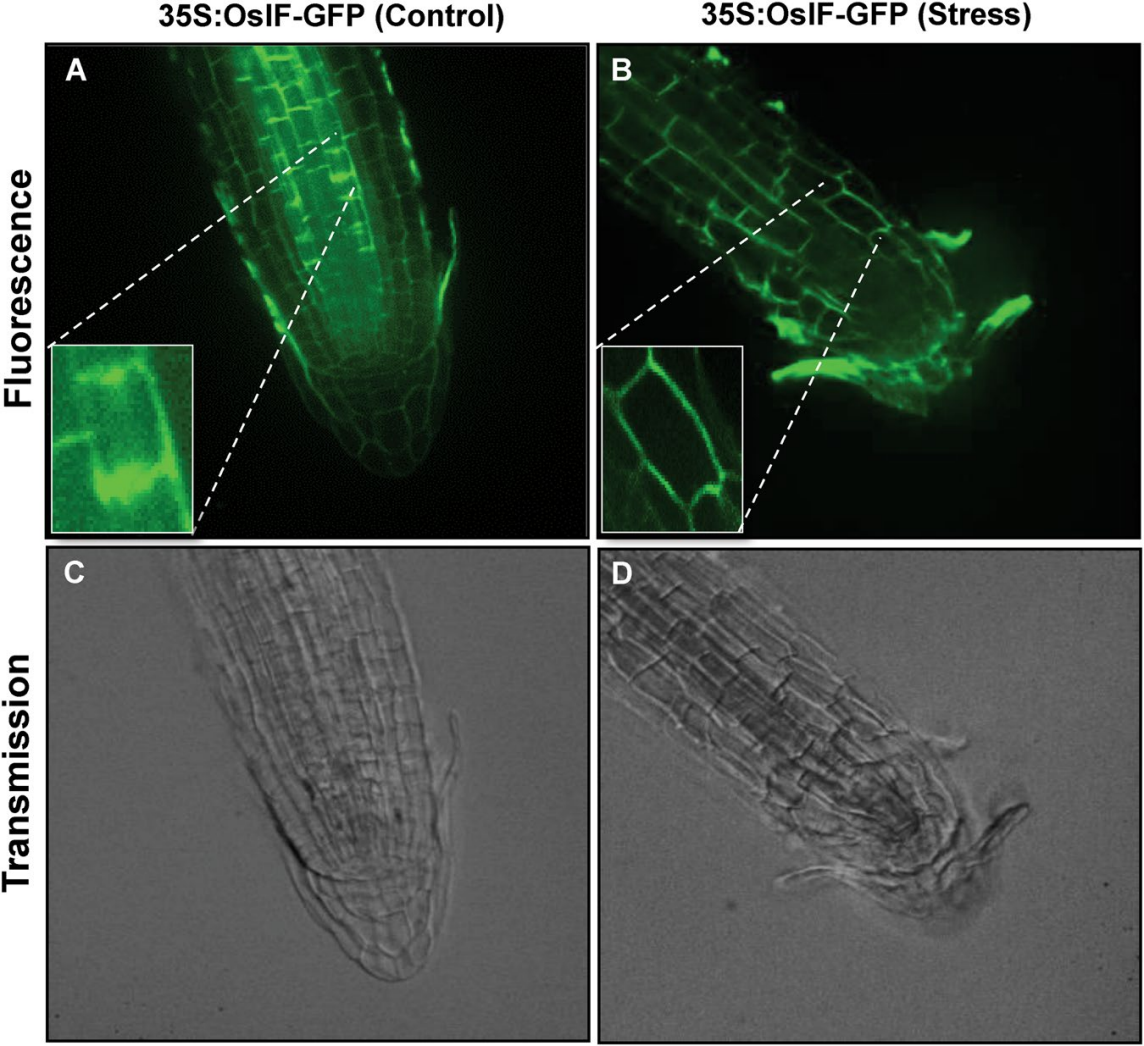
Sub-cellular localization studies of GFP fused OsIF protein in root cells by confocal microscopy. The full-length ORF of OsIF was fused “in-frame” with GFP in pCAMBIA1304 plant expression vector. The construct was used to raise stable transgenic rice plants. GFP fluorescence in 35 S:OsIF-GFP expressing root cells (**A**) untreated and, (**B**) treated with 200 mM NaCl for 30 min in half-strength Yoshida medium (see Methods). Fluorescence in 35 S:OsIF-GFP transformed roots was observed in cytosol and in the membranes under non-stress conditions, which was redistributed to cell margins after stress treatment. (**C**) and (**D**) are transmission images.

To study subcellular localization, GFP fluorescence of *OsIF*-GFP fusion protein was observed in roots of transgenic seedlings which were either untreated (control) or treated with salinity. Under control conditions, IF protein was distributed throughout the cells of roots of the transgenics (Fig. 2A and C), but after salt treatment, this protein moved to the cell margins, as observed via GFP fluorescence (Fig. 2B and D). This suggests the presence of intermediate filaments (OsIF) throughout the cell under control conditions, whereas within 30 min of salt stress, intermediate filaments reorient towards the cell margins, possibly to maintain the cell shape under stress. In summary, these observations clearly demonstrate that salinity stress induces reorganization of intermediate filaments.

### OsIF overexpression supports cell survival under salinity stress in rice

The overexpression (OEIF) and knock-down (KDIF) lines of *OsIF* were raised in IR64 background. Southern hybridization of WT and OEIF lines (Supplementary Fig. S2D-E) using the gene specific probe showed two bands in both the overexpression lines (OEIF1 and OEIF2), one for the integrated transgene and other for the endogenous gene (Supplementary Fig. S2E). As expected, only single band corresponding to the endogenous *OsIF* gene was detected in the WT plants (Supplementary Fig. S2E). Comparatively higher expression of *OsIF* transcripts in both the OEIF transgenic lines was observed by qRT-PCR analysis, while the knock-down line (KDIF) showed much less expression of *OsIF* than the WT (Supplementary Fig. S2F). Altogether these results suggest successful overexpression and knock-down of the *OsIF* gene in OEIF and KDIF lines, respectively. After obtaining confirmed transgenic plants for overexpression and knock-down of OsIF, their relative tolerance to the salinity was studied at the seed germination as well as at the mature plant level, and the results are presented below.

### Over/under-expression of *OsIF* affects seed germination and seedling growth of rice under salinity stress

**Figure 3:**
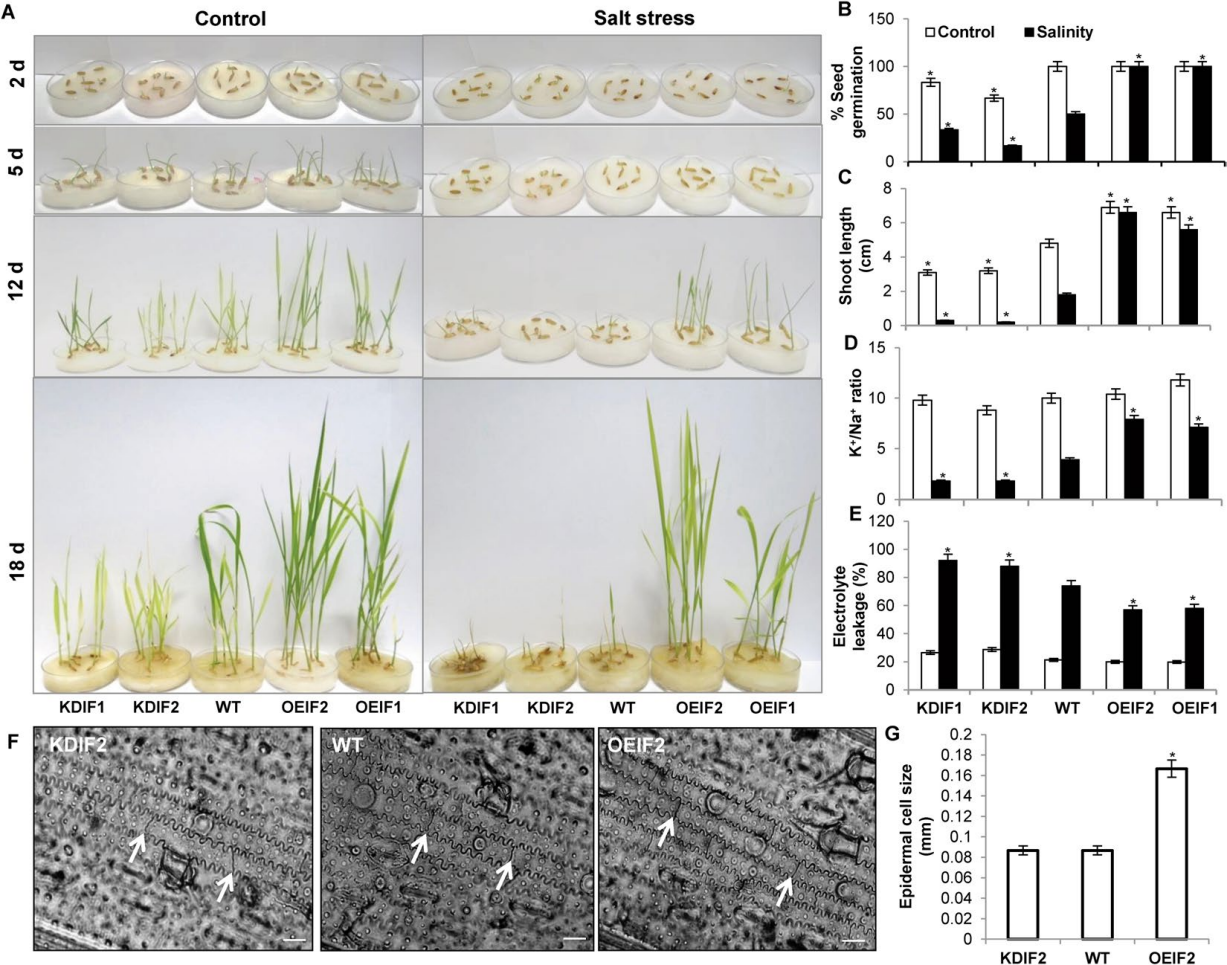
Germination and stress tolerance assay indicated better physiology of over-expression transgenic seedlings (OEIF) than the wild-type (WT) and knock-down (KDIF) seedlings under control (white bars) and salt stress condition (black bars). (**A**) Seed germination and seedling growth assay, under control and salt stress conditions. Pictures were taken at 2, 5, 12 and 18 days of germination; (**B**) Seed germination percentage; (**C**) Shoot length; (**D**) K^+^/Na^+^ ratio; (**E**) Electrolyte leakage percentage as measured for WT, OEIF and KDIF lines; (**F**) Microscopic imaging of 20 d old leaf tissue (epidermal peel) of transgenic and WT plants revealed comparatively elongated epidermal cells in OEIF shoots than in the WT and KDIF (marked by white arrows). (**G**) Measurement of epidermal cell length from the panel. Data are shown as mean ± SE, which were calculated from three independent experiments and the significant difference is shown as [(*)p < 0.05 probability levels] after comparison of WT control with OEIF and KDIF control and WT (salt-stressed) with OEIF and KDIF (salt-stressed). Bar = 0.01 mm.

The efficiency of germination in the presence of high salinity is one of the best parameters to look at salt tolerance of transgenic plants. For this analysis, seeds of WT (raised through tissue culture from untransformed calli, as null event), OEIF and KDIF were germinated under control conditions as well as in the presence of 200 mM NaCl (Fig. 3A). Both the overexpression lines (OEIF1 and OEIF2) performed better than the WT and the knock-down lines (KDIF1 and KDIF2). Significant difference in the seed germination and growth rate was observed even under control conditions, which was more evident under stress at 18 days after germination (DAG). Overexpression of *OsIF* supported better (~98% efficiency) seed germination under salinity stress; in contrast, it was only ~50% in WT, and ~25–40%, in KDIF lines (Fig. 3B). Effect of high salt (200 mM NaCl) was less severe on shoot growth of OEIF seedlings than on the WT and KDIF seedlings (Fig. 3C); difference in the shoot length was evident just after 10 DAG, but became very obvious after 18 DAG.

Although we have not observed any significant differences in K^+^/Na^+^ ratio in all the three types of seedlings under non-stress conditions, yet, salt stress significantly reduced K^+^/Na^+^ ratio in the WT and the KDIF seedlings. Consistent with its better salt tolerance ability, OEIF seedlings showed ~50% and 77% higher K^+^/Na^+^ ratio than the WT and the KDIF seedlings, respectively, after salt treatment (Fig. 3D).

Similarly, effect of high salt was also observed on the electrolyte leakage in the overexpression and the knock-down transgenics, as well as in the WT seedlings. Electrolyte leakage from the OEIF seedlings was ~60%, which was less than from the WT seedlings (~80%). The KDIF seedlings showed maximum membrane damage (~90%) under salt stress, which is consistent with its increased salt sensitivity due to knockdown of the *OsIF* gene (Fig. 3E).

*In summary, all our results show that OEIF transgenic seedlings are better adapted to tolerate salinity stress, than the WT and the KDIF seedlings*. For most of our measurements, the OEIF2 line performed better than the OEIF1. One of the reasons for this difference in tolerance level must be the higher expression of *OsIF* gene in the OEIF2 line (Supplementary Fig. S2F). We selected KDIF2 line for further detailed characterization as differences in physiological parameters were more evident in this line than in the KDIF1 line.

Since we have observed significant elongation of shoots of OEIF transgenic seedlings under control conditions as compared to the WT and KDIF, microscopic examination of epidermal peels of shoots of all the three types of (OEIF2, KDIF2, and WT) seedlings was carried out. These studies showed the presence of comparatively longer epidermal cells in the OEIF2 seedlings than in the WT and the KDIF2 seedlings (Fig. 3F,G), substantiating the increased shoot length as observed in the OEIF transgenic seedlings. These results clearly establish the role of *OsIF* in enabling the seeds to germinate and grow better under saline conditions.

### Effect of salinity stress on transmittance changes at 820 nm and chlorophyll *a* fluorescence transient in WT and transgenic plants

**Figure 4:**
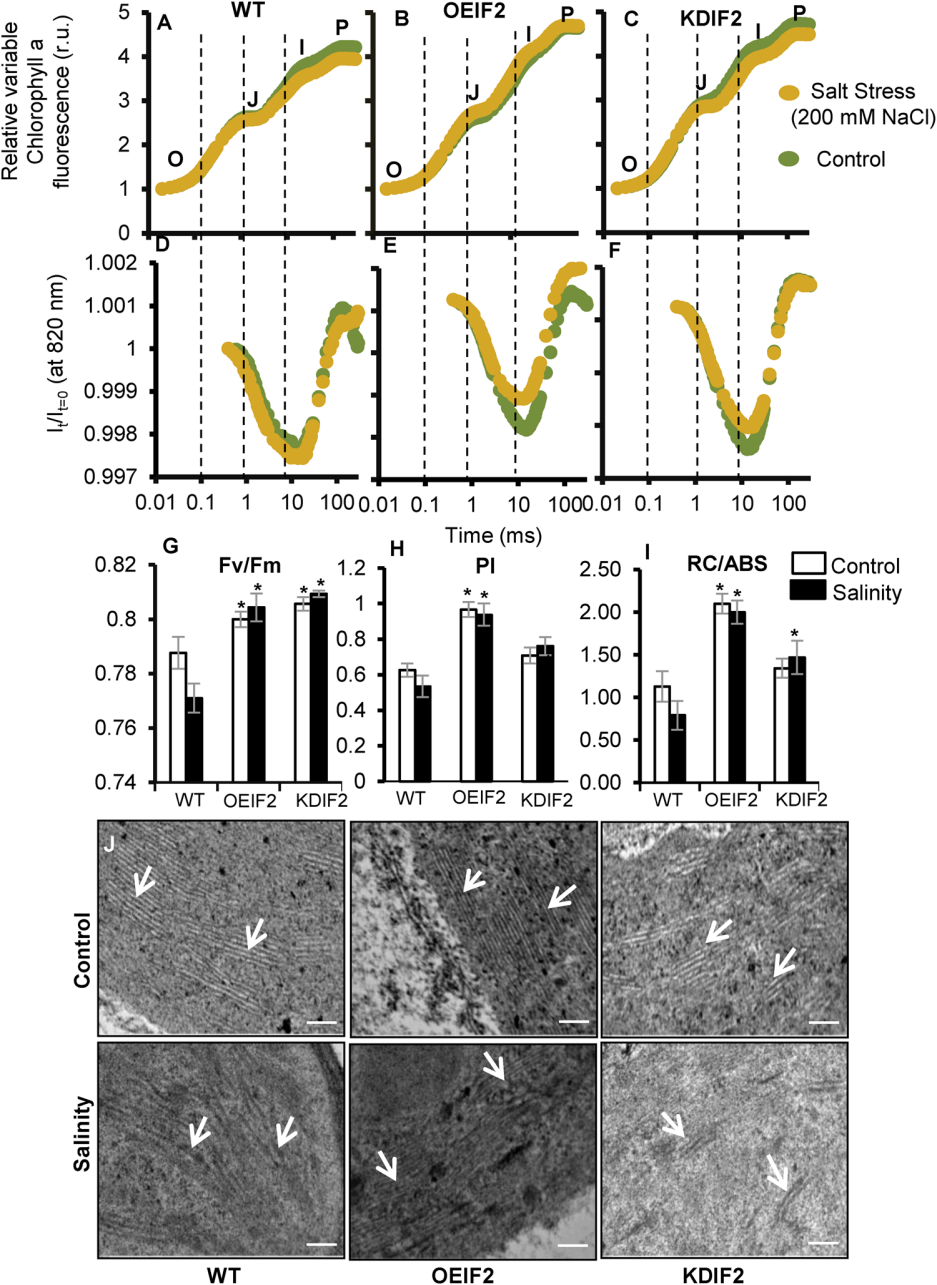
Effect of salt stress on chlorophyll a fluorescence, 820 nm transmittance and grana integrity in the chloroplasts of rice leaves. (**A**–**C**) Simultaneously-measured chlorophyll a fluorescence induction curves; (**D**–**F**) 820 nm transmittance curves in WT, OEIF2 and KDIF2 rice plants under control and salinity stress. The curves are average of six different samples. (**G**) Fv/Fm; (**H**) PI (Performance Index); (**I**) RC/ABS, which is related to the number of active PSII reaction centers per PSII antenna Chl; (**J**) Ultrastructure of chloroplasts of WT, OEIF2 and KDIF2 lines, without salt (control), and after 24 h of salt (200 mM NaCl) stress, showed more disintegration of chloroplast grana in WT and KDIF2 shoots under stress than in OEIF2. Data are shown as mean ± SE, calculated from three independent experiments and the significant difference is shown as (*)p < 0.05 probability levels as compared to the WT under control and stress conditions. Bar = 200 nm.

Results presented thus far clearly demonstrate that overexpression of *OsIF* in rice increases its tolerance to salinity stress (Fig. 3). This enhanced tolerance of OEIF2 was supported by changes in the fast (until 1 s) chlorophyll (Chl) *a* fluorescence induction curve (also called the OJIP transient, where O is the origin, J and I are two intermediary inflections, and P is the peak (Fig. 4A–C, see below for details^7–9^). However, the 820 nm transmittance (that is mostly due to changes in the redox state of P700, the primary electron donor of PSI^10,11^) shows the following result: in OEIF2, salt increased this transmittance, whereas in WT it showed no such effect, but KDIF2 was intermediate, indicating that salt increased the oxidation of P700 (i.e. PSI reaction center) more in OEIF2, than in the wild type (Fig. 4D–F; Supplementary Tables S1 and S2). Further, we also note that in both control and salt-treated samples, the transmittance at 820 nm is lowest in OEIF2, followed by KDIF2 and then the WT (Supplementary Fig. S3A and B).

We now discuss the effect of 200 mM NaCl on Chl *a* fluorescence changes. Differences between the OJIP transients of control and salt treated WT, OEIF2 and KDIF2 transgenic plants are shown in Fig. 4A–C (also see: Supplementary Fig. S3C and D). In salt treated plants, as compared to their control, we observe: (i) 6.4% decrease in maximum fluorescence (the P level; Fm) in WT, whereas no change was observed in OEIF2 plants; however KDIF2 had a 4.8% decrease in the P level; (ii) a higher maximum fluorescence (Fm) for OEIF2 plants under stress than in WT (by 19%) and KDIF2 (by 4.5%), as well as higher maximum variable fluorescence (Fv = Fm − Fo, where Fo is the minimal fluorescence) for OEIF2 (25.4% and 5.7% than in WT and KDIF2, respectively); in WT and KDIF2, Fv decreased by 8.4% and 6%, but in OEIF2 it showed no change. (iii) slower initial O-J fluorescence rise (calculated as dV/dto, where V_t_ = (F_t_ − Fo)/Fv is the relative variable fluorescence at time “t”; see Methods) in OEIF2 than in WT and KDIF2 (Supplementary Fig. S3E and F; Supplementary Table S2); (iv) lower J and I steps in OEIF2, as compared to the WT and KDIF2 (see double normalized curves in Supplementary Fig. S3E and F); and (v) a larger area over the OJIP curve in OEIF2, than in the WT and KDIF2 (Supplementary Fig. S3E and F).

Analysis of differences between WT, OEIF2 and KDIF2 plants, by using Fv/Fm, which is a measure of the quantum yield of photosystem II photochemistry, the parameter RC/ABS, which evaluates the number of active PSII reaction centers, and the “performance index” PI (abs), which evaluates the photosystem II performance (see Methods, and a review by Stirbet *et al*.^12^) led to the following results and conclusions. In OEIF2 and KDIF2 plants, as compared to WT plants, Fv/Fm, and thus the maximum quantum yield of PSII photochemistry, was slightly higher (by 3%) (Fig. 4G) under control conditions, but in all the samples (control and salt-treated) it had values that ranged between 0.77 and 0.81, showing that they had almost fully functional photosynthesis^12^. As we already know, salt-stress has a more pronounced inhibitory effect on the WT plants (Fig. 3A–F), whereas the OEIF2 plants are tolerant to it; Fv/Fm in the WT plants decreased by ~3%, whereas in OEIF and KDIF plants, there was no significant change (~+0.4%) (Fig. 4G). The observed slower initial O-J rise in the OEIF2 plants suggests lower functional PSII antenna size^7,8^ than in the WT and in the KDIF2 (Supplementary Fig. S3E and F)^13^. The I-P phase, which is strongly influenced by PSI activity^14^, is also different in the OEIF2 plants from that in the WT and KDIF2 plants: in the OEIF2, it started from a higher I-level, and increased to the P-level faster than in the WT (Supplementary Fig. S3C and D)^11^. We note that higher I-level (in double normalised fluorescence transients), as noted above, has been suggested to be related to lower PSI/PSII ratio^15^.

Finally, PI (abs) in OEIF2 and KDIF2 plants was much higher (by >100% and ~86%, respectively) compared to the WT plants under non-stress conditions, indicating their higher potential for a better photosynthetic performance. PI (abs) decreased in WT and OEIF2 plants by ~30% and 5%, respectively, when they were treated with high salt, whereas in KDIF2 plants it increased by ~10% (Fig. 4H). Results on RC/ABS (inverse of antenna size) are also in agreement with Fv/Fm and PI data (Fig. 4I–H).

In conclusion, all of the above suggest that OEIF2 plants are superior, as compared to WT, in their performance under salt stress, as monitored by several independent measurements in this study (see the above list and Figs 3 and 4), confirming their increased tolerance to salt treatment, described above.

Figure 4J shows transmission electron microscopic (TEM) images of 7d old leaves of WT, OEIF2 and KDIF2 seedlings before and after treatment with 200 mM NaCl. These images show disorganization of grana stacks in the chloroplasts of WT and KDIF2 leaves after 24 h of salt treatment, whereas the ultrastructure of OEIF2 chloroplasts had only a very slight effect of salt stress (Fig. 4J).

### Overexpression of OsIF provides salinity and heat stress tolerance to transgenic rice plants at the reproductive stage

**Figure 5:**
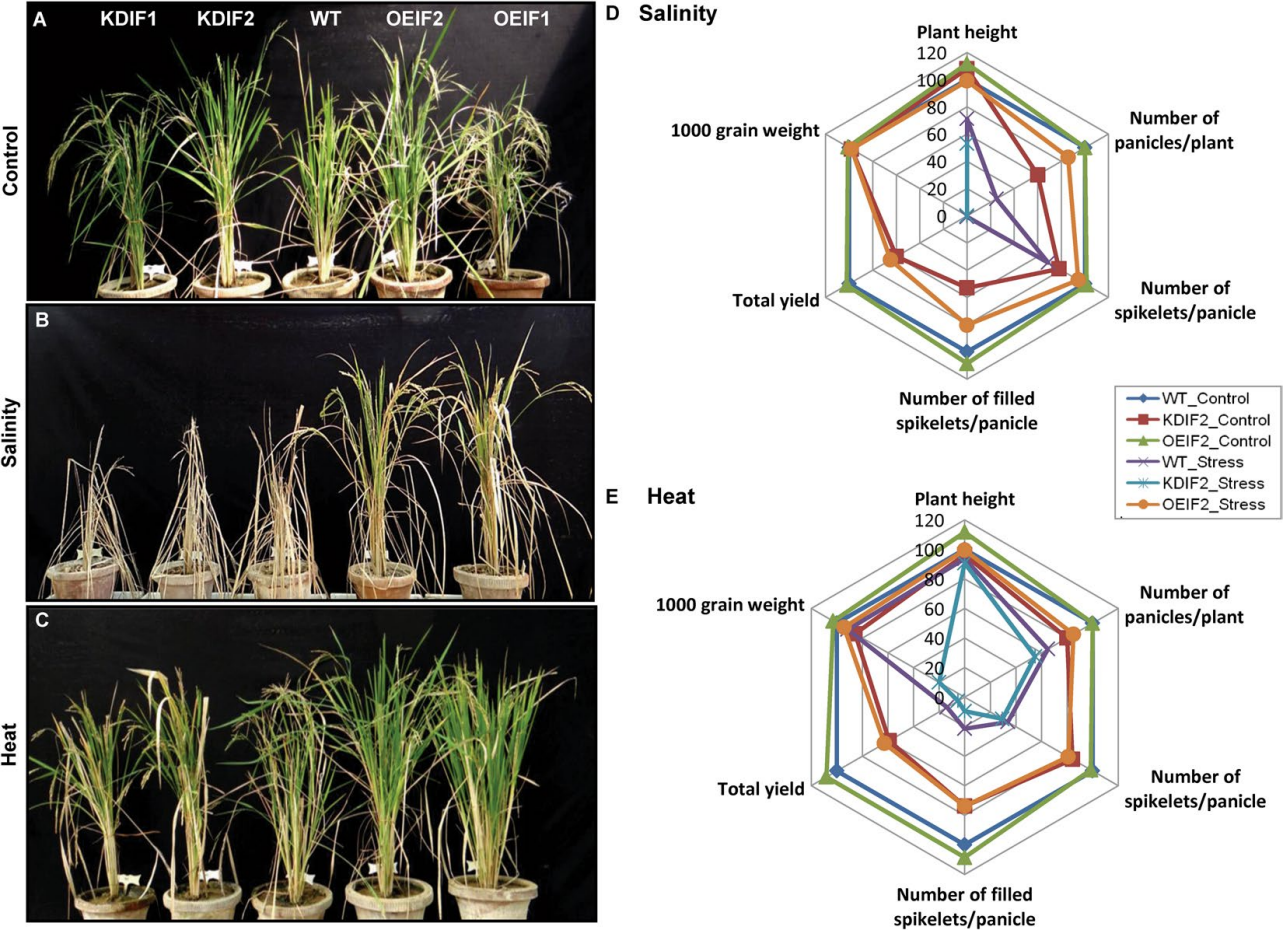
Relative salinity and heat stress tolerance assay of WT, OEIF and KDIF transgenic rice plants. WT, knockdown (KDIF1 and KDIF2) and over-expression (OEIF2 and OEIF1) transgenic lines under (**A**) Control; (**B**) Salinity (150 mM NaCl); and (**C**) Heat (40 °C day/35 °C night temp) stress imposed at pre-flowering stage for 15 days and then recovered till maturity to evaluate the effect of stress on its yield. Picture was taken after 45 days of recovery. Various yield related parameters were evaluated in the WT, KDIF and OEIF transgenic plants under control as well as under high salinity (**D**) and under heat stress (**E**) condition, and are presented in the form of a web diagram.

Earlier studies (based on the qRT-PCR data, as well as on the above experiments), at the seedling stage, suggest that *OsIF* may be involved in heat and salt stress tolerance^6^. We speculate that increased (or reduced) OsIF abundance must also influence plant stress response at the reproductive stage. To test this hypothesis, the stress tolerance of both OEIF2 and KDIF2 transgenic rice lines at their reproductive stage was examined (Fig. 5A–C). We exposed WT, KDIF2 and OEIF2 transgenic rice plants of the same age (and visually of similar physiological growth) to high salt (150 mM NaCl) or heat stress (42 °C), until visible stress symptoms such as leaf yellowing appeared. Plants irrigated with water, grown at 28 °C, were used as experimental control. Under control conditions, we did not observe any major differences in the growth of WT and transgenic plants (Fig. 5A). However, salt stress imposed severe growth constraints on KDIF2 transgenic lines (Fig. 5B). These plants could not survive under 150 mM NaCl and did not reach their reproductive phase (Fig. 5B and D). Though lesser than the KDIF2 plants, WT also showed yellowing of their tillers. WT plants showed 70% less panicles and 27% less spikelets per panicle than the OEIF2 plants under salt stress which show better survival and seed set under these conditions (Fig. 5B and D). In response to heat stress, KDIF2 plants could complete their life cycle, but they had reduced vegetative and reproductive growth (Fig. 5C and E). Both WT and KDIF2 plants had less number of panicles (23% and 35% of OEIF, respectively) with lesser filled grains (70% and 87% of OEIF2, respectively), which in turn led to drastic reduction in the yield of WT (77%) and the KDIF2 (91%) than in the OEIF2 line (Fig. 5E; Table 1). Web diagrams, representing yield data under stress, show better performance and yield of salt stressed OEIF2 lines than the KDIF2 and the WT plants (Fig. 5D and E). Similarly, under heat stress, OEIF2 plants gave almost equal yield as that obtained from WT plants under control conditions. The data for each of the agronomic parameters is provided in Table 1. These results suggest direct correlation of OsIF with stress tolerance phenotype in the transgenic lines.

**Table 1:** Yield related parameters for wild-type (WT), over-expression (OEIF2) and knock-down (KDIF2) plants under control, salinity and heat stress conditions.

	Control			Salinity stress			Heat stress		
	WT	KDIF2	OEIF2	WT	KDIF2	OEIF2	WT	KDIF2	OEIF2
Plant Height (cm)	68.3 ± 0.53	73.7 ± 032	76.3 ± 0.18	48.6 ± 0.32	36.1 ± 0.48	67.6 ± 0.16	64 ± 1.6	62 ± 0.4	68 ± 1.2
Number of Panicle/plant	20 ± 1.63	12 ± 0.81	20 ± 3.63	5 ± 0.81	0	17 ± 0.816	13 ± 1.62	11 ± 0.8	17 ± 0.81
Number of Spikelets/panicle	118 ± 1.72	92 ± 3.26	117 ± 3.52	81 ± 2.4	0	112 ± 4.8	40 ± 3.2	35 ± 0.8	96 ± 2.4
Number of filled spikelets/panicle	84 ± 3.26	44 ± 0.82	91 ± 2.63	1.2 ± 0.16	0	67 ± 2.4	18 ± 1.6	8 ± 0.8	62 ± 0.81
Total Yield (total number of filled grains per plant)	1680 ± 65.3	528 ± 6.53	1816 ± 10.8	3 ± 0.81	0	1155 ± 4.08	234 ± 3.3	88 ± 6.4	1054 ± 3.2
1000 grain weight (gm)	35 ± 0.81	30 ± 0.82	36 ± 1.8	32 ± 0.4	0	33 ± 0.42	32 ± 0.5	28 ± 0.4	33 ± 0.38

### Wild type and OsIF overexpression seedlings show distinct metabolic response under salinity stress

To investigate the physiological mechanism of salt tolerance in the overexpression transgenic lines, we carried out time dependent metabolic profiling of OEIF2 and WT seedlings exposed to 200 mM NaCl. In the chromatographic analysis, we observed reproducible and stable differences between the samples, indicating the reliability of the analysis. A total of 523 types of metabolites (159 known and 364 unknown metabolites) were identified and their relative concentrations were determined (Supplementary Table S3).

The distribution intensity of metabolites before and after the normalization steps is shown as Box-whisker plots in the upper panel of Supplementary Fig. S4, whereas, linear distribution plots, prior and after normalization of data are shown in the lower panel of Supplementary Fig. S4. After normalization, the data show a typical Gaussian distribution (see the log-transformed curve on the right), confirming the good quality of the data. The samples of control and salt treatments of different durations were separated by PC2 (Principal Component 2) which represented 17.5% of variations among the samples. PC1 distinguished WT samples from OEIF2 transgenic samples, explaining 27.4% of the observed variations (Supplementary Fig. S5). This score plot clearly shows the “separation” of the WT metabolome from the transgenic OEIF2, under both non-stress and stress conditions. Further, metabolic profiles at various time points did not fluctuate much in the wild type, even under stress conditions, whereas, in the transgenic seedlings, metabolic variations were pronounced.

**Figure 6:**
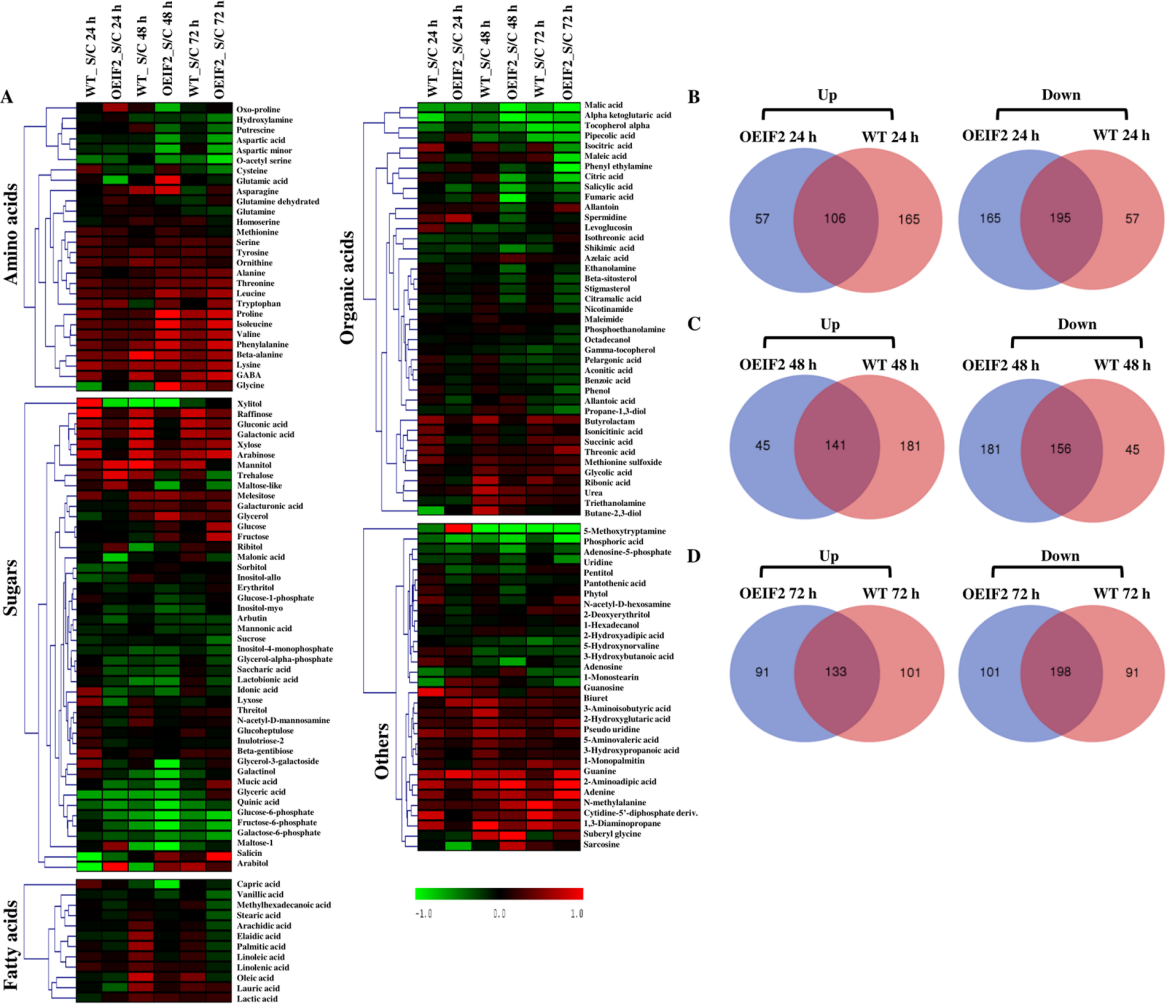
Determination of metabolites in the wild type (WT) and in the OsIF over-expressing (OEIF2) transgenic lines, using gas chromatography-mass spectrometry. (**A**) Heat map of wild-type (WT) and OsIF over-expressed (OEIF2) transgenic lines showing differential accumulation pattern of various metabolites at 24 h, 48 h and 72 h time points of log_2_ value of fold change after calculating the ratio of stress and control (S/C) parameters. Venn diagrams showing increased (up) and decreased (down) metabolites between OEIF2 and WT at 24 h (**B**), 48 h (**C**) and 72 h (**D**) of salinity stress.

Heat-map generated from log_2_ of fold change values (stress/control) at different time points from the WT and the OEIF2 transgenic seedlings suggests that there may have been a significantly large reprogramming of the metabolome in both the WT and the OEIF2 transgenic seedlings in response to salinity stress (Fig. 6A). Striking differences were observed in temporal metabolite profiles of the WT and OEIF2 transgenic seedlings. Increased abundance of most of the stress related metabolites were observed during both the early as well as the late stress durations in the transgenic seedlings than in the WT. Changes in quantity of osmolytes, such as GABA and proline, were less in OEIF2 seedlings at 24 h which, however, further increased with increase in stress duration. While in WT, comparative abundance of these metabolites was high at 24 h, which decreased with increasing stress treatment. Sugars (e.g., glucose and fructose) increased in OEIF2 seedlings with the treatment time. In OEIF2, we have observed ~6–7 fold higher accumulation of proline than in the WT seedlings under salt stress, especially after 48 h of stress (Supplementary Fig. S6). In contrast to this, stress led to decreased abundance of these sugars in the WT (Supplementary Fig. S6). We also observed less accumulation of phenolic compounds in the transgenic seedlings, under stress, than in the WT (Fig. 6).

Stress induced increase in free amino acids (e.g., phenylalanine, isoleucine, valine, alanine, tryptophan, proline, gamma amino butyric acid (GABA), threonine and leucine), inositol, oxoproline, glucose, fructose, and glycine was also observed in OEIF2 seedlings (Supplementary Fig. S6). One of the notable features of this analysis was that the OEIF2 line, as compared to the WT, had constitutively higher amounts of all the amino acids in control samples corresponding to 24 h salt stress treated samples. These amino acids showed increased accumulation in both the WT and the transgenic seedlings in response to salinity stress.

Overall accumulation of osmolytes, such as complex sugars (raffinose, trehalose and mannitol) was higher in OEIF2 seedlings as compared to WT under stress (Supplementary Fig. S6). Similarly, the transgenic seedlings also showed increased accumulation of sugars, such as glucose, and fructose, as well as their derivatives, than the WT seedlings under stress. Metabolic profiles of WT and transgenic seedlings suggest better stress regulatory machinery in OEIF2 seedlings, since they maintained higher abundance of most of the osmolytes (e.g., proline and mannitol), TCA cycle components and free amino acids.

## Discussion

Though several research groups, worldwide, are involved in the understanding of the complex mechanism of abiotic stress tolerance in plants, it has been very difficult to pin down a single candidate gene or mechanism for improving the survival of plants, as well as their yield under stress. This may be due to the fact that abiotic stress tolerance is a quantitative trait, which is also regulated by a combination of several environmental factors. Further, this may lead to an increase in expression of different stress related genes, which, in turn, regulate stress responses either by a common pathway or through different interconnected pathways^16^. In our study here, we report multi-stress (salt, drought, high temperature, heavy metal, methyl viologen and abscisic acid) inducible changes in *OsIF* transcript levels, suggesting them to be members of cell stress response machinery (Fig. 1A–F).

Analysis of transcript accumulation in seedlings of salt-sensitive rice genotype IR64 and salinity-tolerant genotype Pokkali has revealed *OsIF* to be differentially regulated in two contrasting genotypes. As compared to IR64, Pokkali shows constitutive higher transcript abundance of *OsIF* (Fig. 1A–F). Several other investigators^5,17–19^ have also shown higher abundance of stress related transcript and proteins in tolerant genotypes than in sensitive genotypes under non-stress conditions, thereby suggesting pre-preparedness of tolerant genotypes to deal with stress. Further, several researchers^20^ have suggested interaction of IF proteins with heat shock proteins (HSPs), indicating their possible role in high temperature tolerance; in this connection, we have also reported a drastic increase in transcript abundance of *OsIF* under heat stress (Fig. 1C), suggesting towards a putative role of intermediate filaments in plant heat stress response.

Reorientation of cytoskeleton (CSK) components in the presence of high salts is another cell survival response in plants^21^. OsIFs also undergo salt stress induced reorganization (Fig. 2), which has also been reported for other CSK components, i.e., microtubules (MTs) and microfilaments (MFs)^21^. Figure 2 shows that salt treatment led to plasmolysis of cells, where GFP fluorescence was seen only at the cell margins, suggesting reorganization of IFs to cell surface to protect cellular integrity. These results also suggest that salt stress induced reorganization of MT array may lead to a collapse of IF array which could be a salt stress response of cells, supporting cell survival under stress conditions^22–24^.

A cellular K^+^/Na^+^ ratio is an indicator of cell toxicity. OEIF transgenic seedlings could maintain higher K^+^/Na^+^ ratio in shoot tissues than WT and KDIF seedlings under salt stress (Fig. 3D) indicating the role of OsIF in preventing ion toxicity. OsIF may influence ion accumulation or transport by interacting with ion transporters and plasma membrane ATPases^6,25^. Higher K^+^/Na^+^ ratio in OEIF plants supports better photosynthetic performance, as increase in Na^+^ content drastically reduces chlorophyll a fluorescence in chloroplasts^26^. Chloroplasts of OEIF2 transgenic seedlings also showed the least effect of salts, in contrast to those from the WT and the KDIF2 seedlings. However, the latter two showed comparatively dilated grana under salt-stress than under non-stress conditions (Fig. 4J). This much more protected chloroplast ultrastructure in OEIF2 plants under salt stress can again be directly correlated with their higher PSII efficiency as compared to those of WT and KDIF2 plants, leading to a higher “performance index” under salt stress conditions (Fig. 4). Further, our results are consistent with the conclusion of Hamdani *et al*.^11^ who had observed a good correlation between the faster IP rise, as well as a higher P level, during Chl a florescence transient (see Fig. 4) with higher biomass in elite, over landrace, rice varieties.

Abiotic stresses such as high temperature and salinity have been well documented to affect yield in cereals including rice^1^. Even one degree rise in temperature at the time of anthesis has been shown to affect pollen viability^27^. In our study, we had subjected rice plants at pre-flowering stage (when formation of reproductive structure begins) to high temperature for 15 days. This set of conditions results in 85% decrease in yield in WT plants thereby indicating detrimental effect of the heat stress imposed on yield. In contrast, the transgenic plants overexpressing OsIF showed only 38% decline in the yield. Similarly the KD plants, where OSIF had been knocked down, showed a drastic phenotype where 95% decrease in the yield was documented under similar set of conditions. Taken together, our study clearly shows the role of OsIF in tolerance towards salinity and high temperature in both the seedling and the reproductive stage. Substantial evidence exists in the literature where sensitivity to salinity and other abiotic stresses has been well documented at the two most sensitive stages, i.e. seedling and reproductive stage of rice^1^.

The ability of plants to maintain a reasonable photosynthesis rate under environmental stress is fundamental for the maintenance of plant growth and development^28^. The finding that the “performance index” (calculated based on Chl a fluorescence data; see Methods and Stirbet *et al*.^12^) in OEIF2 transgenic plants grown under salt stress conditions was better than even in the unstressed WT plants indicates high efficiency of the photosynthetic machinery in the OEIF transgenics. Taken together, these results suggest that OsIF is an integral member of plant stress response machinery. Further investigation is needed to dissect out the direct role of *OsIF* in regulating stress tolerance, and overall photosynthesis capacity.

Plants alter their metabolism in various ways to cope with stress induced altercations. These changes include production of compatible solutes to stabilize proteins, cellular structures and maintenance of cell turgor by osmotic adjustment. Plants remove excess ROS for re-establishing the cellular redox balance^29–32^. We report here a higher steady-state pool of many stress-responsive metabolites like proline, sugars, amino acids, in OEIF2 transgenic seedlings, even before exposure to salinity, conferring higher tolerance to transgenic plants (Supplementary Table S3). Proline is one of the well-known compatible osmolytes, ROS scavenger, and a molecular chaperone stabilizing the structure of proteins, thereby protecting cells from damage caused by stress in transgenic seedlings^33–35^. The role of sugars, such as, trehalose, sucrose, and raffinose, as compatible solutes, has also been reported in response to salt, drought and temperature stress^36–41^. Raffinose has also been implicated in membrane protection and radical scavenging^42,43^. Comparatively higher accumulation of polyols such as mannitol, sorbitol, and inositol, in OEIF2 lines, has also been correlated with stress tolerance in plants^44–47^. There is an obvious time-dependent difference in the metabolite profiles of WT and OEIF2 under control and salt stress (Fig. 6). PLS-DA score plots showed more diverse metabolic profiles of OEIF2 at different time points under control conditions, which in contrast is lesser under stress. This indicates a better energy conservation and stress coping efficiency of the OEIF2 seedlings.

In view of the results, presented in this paper, we speculate that the observed higher accumulation of most of the important compatible solutes in OEIF2 seedlings must help the plant in maintaining osmotic homeostasis and organelle ultrastructure, which, in turn, is responsible for better performance of its photosynthetic machinery. We suggest that the latter, in turn, must be responsible for increased crop yield under salt stress conditions in the transgenics. In the future, live cell imaging studies of OsIF:GFP fusion protein may provide a better insight of their re-orientation under stress conditions and their involvement in protection of cellular machinery.

## Methods

### Plant material and stress treatment

Seeds of IR64 and Pokkali, two well-known rice genotypes with contrasting salt sensitivity^48^, were surface sterilized and germinated in a hydroponic system in half strength Yoshida medium^49^, in a growth chamber at 28 ± 2 °C temperature, 70% relative humidity and 12 h light/12 h dark cycle^19^. Seven-day old seedlings were treated either with different stressors such as, 200 mM NaCl, 500 mM mannitol, 1 μM methyl viologen, 100 μM ABA (abscisic acid), 10 μM CdCl_2_ by adding them to the half strength Yoshida medium. Heat stress was given by increasing growth chamber temperature to 42 °C. Shoot tissues from theses seedlings were harvested at different time points (30 min, 24 h and 48 h), while the seedlings grown in half strength Yoshida medium were used as controls. Plant tissues were collected, frozen instantly in liquid nitrogen, and stored in −80 °C freezer for further studies.

### BLAST search and multiple sequence alignment analysis of OsIF

Proteins homologous to OsIF (LOC_Os01g18840) were identified by Blastp tool of NCBI BLAST (Basic Local Alignment Search Tool) search with a cut-off of e < 10^−5^. Clustal X 2.0 was used for multiple sequence alignment with default parameters^50^.

### Sample preparation, RNA extraction and gene expression analysis

To study the relative transcript abundance of *OsIF*, qRT-PCR technique was used. Total RNA was extracted from the shoot tissues using Trizol reagent (Sigma-Aldrich Inc, USA) and then treated with RNase-free DNase I. Enrichment of polyA^+^ RNA, cDNA synthesis and qRT-PCR was carried out following the protocol published in Soda *et al*.^5^. The primers used for this study are reported in Supplementary Table S4.

### Vector construction and generation of transgenic rice plants

The full length cDNA of the *OsIF* gene (LOC_Os01g18840) was amplified and cloned into pCAMBIA1304 plant overexpression vector (Supplementary Fig. S2A). For knock-down of the *OsIF* gene, its highly specific 200 base sequence was cloned in both sense and antisense orientations in the pFGC1008 vector (Supplementary Fig. S2B). Recombinant plasmids thus obtained were individually transferred into Agrobacterium (strain LBA4404) for the transformation of IR64 rice genotype using calli as explants^51^. The preliminary molecular characterization of putative overexpression transgenic lines was carried out by tissue PCR as done earlier^51^ (Supplementary Fig. S2C). To confirm stable transgene integration, fall out Southern blot analysis was performed (Supplementary Fig. S2,D,E). Genomic DNA extraction, gel electrophoresis, and blotting conditions were performed as described by Sambrook *et al*.^52^. For confirmation of relative expression levels of OsIF in overexpression and RNAi transgenic lines, qRT-PCR analysis was performed (Supplementary Fig. S2F). Overexpression and RNAi lines, thus obtained, were designated as OEIF and KDIF, respectively. The positive plants were then transferred to the greenhouse and grown further. The primers used for this study are reported in Supplementary Table S4.

### Subcellular localization of OsIF

Full length ORF of *OsIF* without stop codon was cloned in pCAMBIA1304 vector in frame with GFP to generate translational fusion of OsIF::mGFP under the control of CaMV35S promoter. The resulting plasmid was used to raise stable transgenic rice plants, as mentioned above. Positive transgenic plants were selected on hygromycin and confirmed by tissue PCR. Roots of transgenic plants at the 4-leaf stage were examined under fluorescence microscope. For salinity stress, plants were given 200 mM NaCl treatment for 30 min in half-strength Yoshida medium.

### Seed germination and seedling growth assay under stress

The seed germination assay was performed in T_2_ generation under salinity stress. Seeds of IF transgenics (OEIF1, 2 and KDIF1, 2) and wild type (WT; tissue cultured raised null event) rice plants were surface sterilized and germinated on MS media with or without 200 mM NaCl in petri-plates. Percent seed germination and seedling growth was measured from 1 to 18 DAG. Shoot length, K^+^/Na^+^ ratio and electrolyte leakage were measured in these seedlings after 18 DAG under stress and non-stress conditions.

### Stress tolerance assays of the wild-type (WT) and transgenic plants

All the physiological analysis was done on both control and 200 mM NaCl treated 18 DAG seedlings, grown in hydroponics system in half Yoshida medium. Measurement of Na^+^ and K^+^ concentrations was done as described by Gupta *et al*.^53^. For the analysis of electrolyte leakage, we used the protocol of Dionisio-Sese and Tobita^54^. WT, knockdown (KDIF1 and KDIF2) and over-expression (OEIF2 and OEIF1) transgenic lines were exposed to salinity (150 mM NaCl); and heat (40 °C day/35 °C night) stress at pre-flowering stage for 15 d and then recovered till maturity to evaluate the effect of stress on their yield. For each parameter, three biological replicates with three technical replicates each (n = 9) were used and standard errors were calculated.

### Epidermal cell size estimation

The size of cells in the epidermal peels from second leaves of 20 day old seedlings of OEIF2 and KDIF2 transgenic rice as well as WT was measured under a compound light microscope. The peels, used here, were obtained (by using forceps) from the adaxial surface of the leaves and then stained by safranin dye for observations under the light microscope.

### Measurement of chlorophyll *a* fluorescence and 820 nm transmittance changes

Wild type and T_2_ generation transgenic plants (OEIF2 and KDIF2) were grown in pots in a glasshouse at 28 ± 2 °C (12 h light/dark). Thirty day old plants were watered with or without salt (200 mM NaCl) for one week. Chlorophyll (Chl) *a* fluorescence measurements were made on intact young leaves, attached to plants, which had been kept overnight in the dark. For Chl *a* fluorescence transients (from 10 μs to 1 s), we used a M-PEA fluorimeter (Plant Efficiency Analyser, Hansatech Instruments Ltd., UK), which allows simultaneous measurement of Chl *a* fluorescence transient (for Photosystem II, PSII) and 820 nm transmittance change (for Photosystem I, PSI). During measurements, a 5 mm diameter area of the leaf was illuminated with 3,000 μmol photons m^−2^ s^−1^ of 650 nm continuous light (provided by an array of 3 light-emitting diodes), as well as by a far-red measuring light (modulated at 3.33 kHz) provided by an OD820 LED (Opto Diode Corp., USA) and filtered to provide 830 ± 20 nm light. An average of six measurements, on fluorescence transients and 820 nm transmittance changes from control and salt-treated leaves was taken for this analysis for the WT, OEIF2 and KDIF2 plants. Chlorophyll *a* fluorescence induction curves were analyzed to give us the results presented in the section “Effect of salt on transmittance changes at 820 nm and chlorophyll *a* fluorescence transient in WT and transgenic plants”. In this paper, we have discussed the following parameters calculated from Chl *a* fluorescence data^7–9^: (i) the Fv/Fm = (Fm − Fo)/Fm, which is a measure of the maximum quantum yield of PSII photochemistry, where Fo is the initial (minimum) fluorescence and Fm is the maximum fluorescence; (ii) RC/ABS = (1 − Fo/Fm)/[4(F300 μs − Fo)/(F_J_ − Fo)], which is a measure of the density of PSII reaction centers, where RC is the number of active PSII reaction centers, ABS is the photon flux absorbed by the antenna of PSII units, F_300μs_ and F_J_ are the fluorescence values after 300 μs and ~2 ms illumination; and the performance index PI (abs) = RC/ABS∙[(Fv/Fm)/(1 − Fv/Fm)]∙[(Fm − F_J_)/(F_J_ − Fo)], which evaluates the performance of PSII activity, where the last term (Fm − F_J_)/(F_J_ − Fo) is related to the efficiency of the electron transfer from the first plastoquinone acceptor of PSII (labeled Q_A_) to the plastoquinone pool in the membrane.

### Transmission electron microscopic analysis

Seven days old seedlings were exposed to 200 mM NaCl for 24 h in hydroponics system supplemented with half Yoshida medium. Shoot tissues were harvested and fixed for 24 h in sodium phosphate buffer, pH 7.2, containing 4% (v/v) glutaraldehyde and 3% (w/v) paraformaldehyde. These samples were rinsed three times (20 min each) in sodium phosphate buffer, pH 7.2 and then post-fixed for 1.5 h in sodium phosphate buffer, pH 7.2, containing 1% (v/v) osmium tetroxide; further, the same samples were subsequently dehydrated first with ethanol as follows: 10 min each with 50%, 70%, 90% and 100% (v/v), and then twice with 100% acetone (15 min each). After dehydration, specimens were infiltrated and embedded in Epon-812 Resin. Ultrathin sections (50–70 nm) were prepared using a Leica EM UC 6ultra-microtome. These sections were mounted on copper grids and stained for 10 min with 4% (w/v) uranyl acetate and lead citrate and examined at 60–80 kV using a Zeiss EVO40 transmission electron microscope.

### Extraction, derivatization and analysis of rice shoot tissue metabolites using gas chromatography-mass spectrometry

Hydroponics grown seven day old rice seedlings were treated with 200 mM NaCl for 24 h, 48 h and 72 h, and then shoot tissues were harvested from both salt treated and control samples at different times and stored at −80 °C after snap freezing in liquid nitrogen. Metabolites were extracted from the shoot tissue (100 mg) and derivatized as described by Roessner *et al*.^55^. Derivatized sample volumes of 1 μL were then injected with a split ratio of 25:1, using a hot needle technique in GC-MS (Shimadzu GCMS-QP 2010 PLUS, Japan) for further generation of chromatograms.

Peak detection and mass spectra deconvolution were performed with Leco Chroma-TOF software v.2.25. The resulting files were processed using metabolomics database. Identified metabolites were reported if present in at least 50% of the total number of samples. Each chromatogram was further “controlled” with respect to the total number of identified metabolites and total peak intensities to ensure that outliers did not confound the subsequent statistical analysis. Metabolite data was normalized using ribitol (internal standard)^56^. Statistical analysis of these normalized data was carried out with the Statistica software (v.9.0. StatSoft, Inc., Tulsa, OK, USA). Multivariate statistical analysis was performed by “unsupervised” principal component analysis (PCA) to obtain a general overview of the variance of metabolic phenotypes in the study, by entering metabolite values without study class assignments (MetaboAnalyst 3.0: a web server for metabolomic data analysis and interpretation). A breakdown ANOVA was used for metabolite analyses. A p value of <0.05 was considered significant. Data distributions were displayed by box–whisker plots, giving the mean value for each category and the standard error as box and whiskers for 1.96 times the category standard deviation to indicate 95% confidence intervals, assuming normal distributions. In addition, supervised partial least-square (PLS) statistical analysis was performed.

### Statistical analysis

Experiments were repeated at least three times. P-values were determined by Student’s t-test (the quantification of stress-related genes expression) or by one-way ANOVA using the protected least-significant difference (LSD) test (the quantification of phenotypic differences).

## Electronic supplementary material

Supplementary Material

Supplementary Table 3
