# Publisher Correction: Rice intermediate filament, OsIF, stabilizes photosynthetic machinery and yield under salinity and heat stress

**DOI:** 10.1038/s41598-018-25624-0

**Published:** 2018-05-11

**Authors:** Neelam Soda, Brijesh K. Gupta, Khalid Anwar, Ashutosh Sharan, Govindjee, Sneh L. Singla-Pareek, Ashwani Pareek

**Affiliations:** 1 Stress Physiology and Molecular Biology Laboratory, School of Life Sciences, Jawaharlal Nehru University, New Delhi, 110067 India; 2 Plant Stress Biology, International Centre for Genetic Engineering and Biotechnology, Aruna Asaf Ali Marg, New Delhi, 110067 India; 3 Department of Biochemistry, Center of Biophysics & Quantitative Biology, University of Illinois at Urbana-Champaign, 265 Morrill Hall, 505 South Goodwin Av, Urbana, IL 61801-3707 USA; 4 The UWA Institute of Agriculture, School of Agriculture and Environment, The University of Western Australia, Perth, WA Australia

Correction to: *Scientific Reports* 10.1038/s41598-018-22131-0, published online 06 March 2018

In the original version of this Article, Ashwani Pareek was incorrectly affiliated with ‘Department of Plant Biology, University of Illinois at Urbana-Champaign, 265 Morrill Hall, 505 South Goodwin Av, Urbana, IL, 61801-3707, USA’. The correct affiliation is listed below.

‘The UWA Institute of Agriculture, School of Agriculture and Environment, The University of Western Australia, Perth, WA, Australia’.

This error has now been corrected in the PDF and HTML versions of the Article.

