# Author Correction: Rice intermediate filament, OsIF, stabilizes photosynthetic machinery and yield under salinity and heat stress

**DOI:** 10.1038/s41598-019-46780-x

**Published:** 2019-07-24

**Authors:** Neelam Soda, Brijesh K. Gupta, Khalid Anwar, Ashutosh Sharan, Govindjee, Sneh L. Singla-Pareek, Ashwani Pareek

**Affiliations:** 1 Stress Physiology and Molecular Biology Laboratory, School of Life Sciences, Jawaharlal Nehru University, New Delhi, 110067 India; 2 Plant Stress Biology, International Centre for Genetic Engineering and Biotechnology, Aruna Asaf Ali Marg, New Delhi, 110067 India; 3 Department of Biochemistry, Center of Biophysics & Quantitative Biology, University of Illinois at Urbana-Champaign, 265 Morrill Hall, 505 South Goodwin Av, Urbana, IL 61801-3707 USA; 4 The UWA Institute of Agriculture, School of Agriculture and Environment, The University of Western Australia, Perth, WA Australia

Correction to: *Scientific Reports* 10.1038/s41598-018-22131-0, published online 06 March 2018

In Figure 3A, the image taken at 5 days of germination under salt stress conditions is incorrect. The correct Figure 3 appears below as Figure 1.

**Figure 1:**
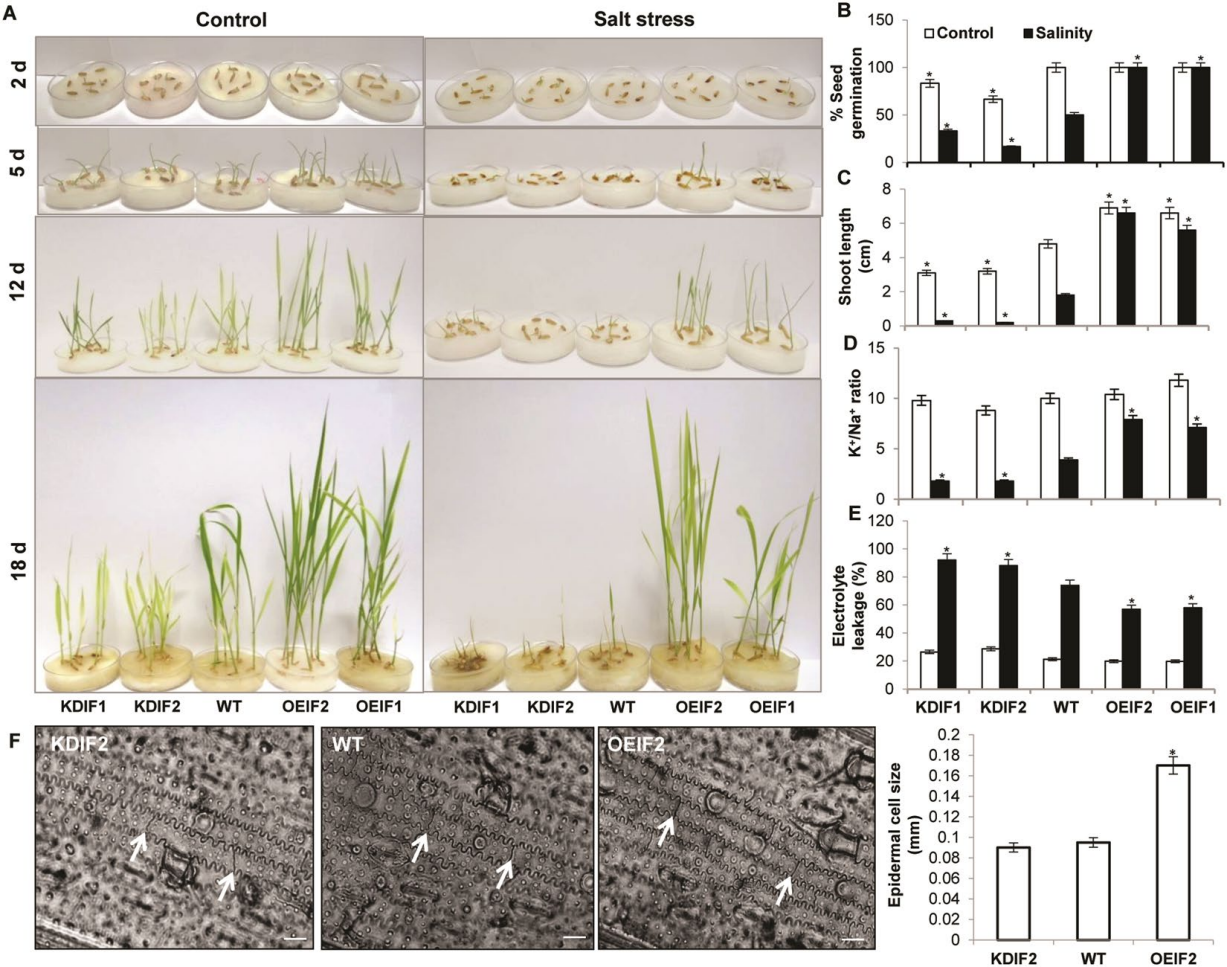
Germination and stress tolerance assay indicated better physiology of over-expression transgenic seedlings (OEIF) than the wild-type (WT) and knock-down (KDIF) seedlings under control (white bars) and salt stress condition (black bars). (**A**) Seed germination and seedling growth assay, under control and salt stress conditions. Pictures were taken at 2, 5, 12 and 18 days of germination; (**B**) Seed germination percentage; (**C**) Shoot length; (**D**) K^+^/Na^+^ ratio; (**E**) Electrolyte leakage percentage as measured for WT, OEIF and KDIF lines; (**F**) Microscopic imaging of 20 d old leaf tissue (epidermal peel) of transgenic and WT plants revealed comparatively elongated epidermal cells in OEIF shoots than in the WT and KDIF (marked by white arrows). (**G**) Measurement of epidermal cell length from the panel. Data are shown as mean ± SE, which were calculated from three independent experiments and the significant difference is shown as [(*)p < 0.05 probability levels] after comparison of WT control with OEIF and KDIF control and WT (salt-stressed) with OEIF and KDIF (salt-stressed). Bar = 0.01 mm.

