# Supplementary material for: Rice intermediate filament, OsIF, stabilizes photosynthetic machinery and yield under salinity and heat stress
Source: Sci Rep. 2018 Mar 6;8:4072. doi: 10.1038/s41598-018-22131-0 (PMC5840354; doi:10.1038/s41598-018-22131-0)
Supplement: Supplementary file 1 — Supplementary Material [file 41598_2018_22131_MOESM1_ESM.pdf]

Title: Rice intermediate filament, OsIF, stabilizes photosynthetic machinery and yield under salinity and heat stress

Neelam Soda[1#], Brijesh K. Gupta[2#], Khalid Anwar[1], Ashutosh Sharan[1], Govindjee Govindjee[3], Sneh Lata Singla-Pareek[2], Ashwani Pareek[1, 4*]

[1]Stress Physiology and Molecular Biology Laboratory, School of Life Sciences, Jawaharlal Nehru University, New Delhi 110067, India

[2]Plant Stress Biology, International Centre for Genetic Engineering and Biotechnology, Aruna Asaf Ali Marg, New Delhi 110067, India

[3]Department of Biochemistry, Center of Biophysics & Quantitative Biology, and Department of Plant Biology, University of Illinois at Urbana-Champaign, 265 Morrill Hall, 505 South Goodwin Av, Urbana, IL, 61801-3707, USA

[4]The UWA Institute of Agriculture, School of Agriculture and Environment, The University of Western Australia, Perth, WA, Australia

# Equal contribution

***Corresponding Authors:**

Ashwani Pareek, Professor
Stress Physiology and Molecular Biology Laboratory, School of Life Sciences,
Jawaharlal Nehru University, New Delhi 110067, India
Email: ashwanip@mail.jnu.ac.in

Supplementary Figure S1:

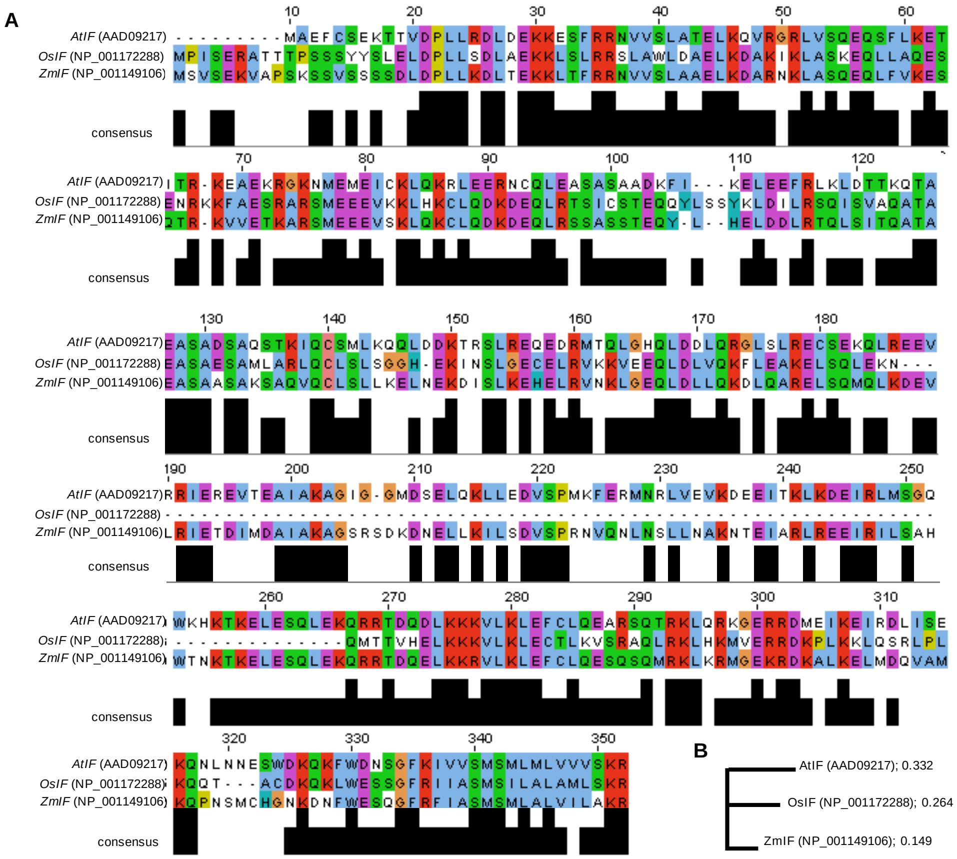

Supplementary Figure S1: Phylogenetic analysis of OsIF in comparison to other known IFs across the genera. (A) Multiple sequence alignment of OsIF with other reported IFs sequences. The Jalview multiple alignment editor was used for multiple sequence alignment. (B) Dendrogram showing approximated phylogenetic distance among IF proteins from diverse genera.

Supplementary Figure S2:

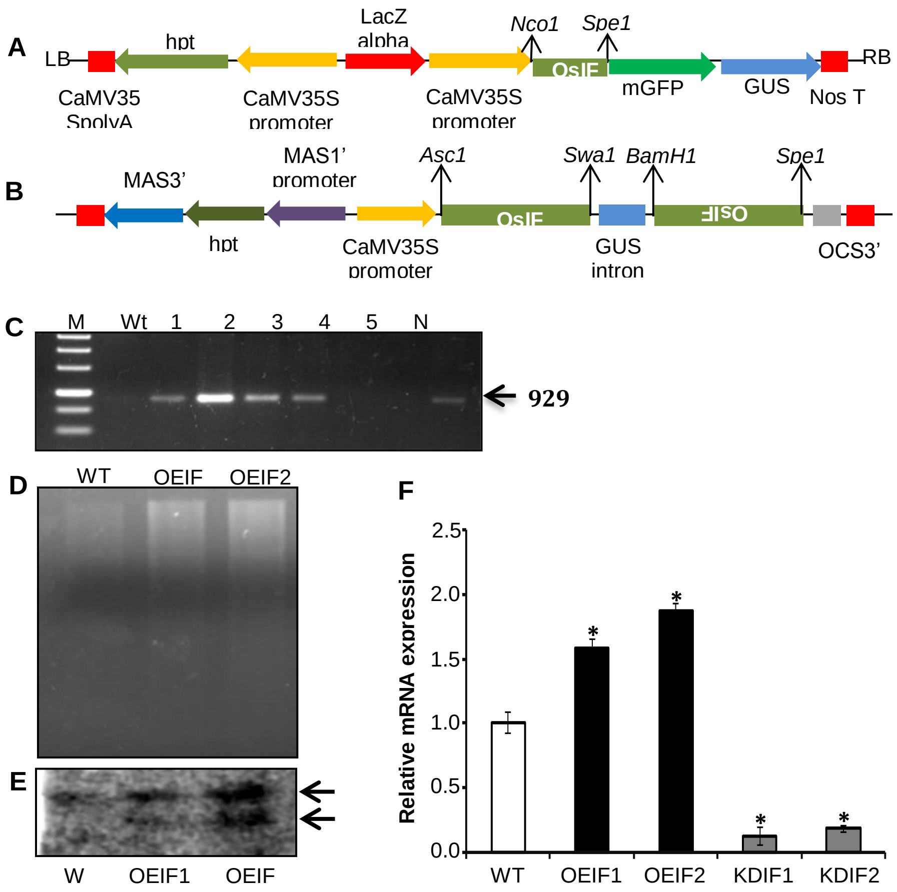

Supplemental Figure S2: Gene constructs used for raising transgenic rice seedlings and their molecular characterization. (A) Gene construct prepared in pCAMBIA1304 for overexpression of OsIF (B) RNAi construct in pFGC1008 for knockdown of OsIF. (C) Molecular confirmation of putative overexpression transgenic lines by tissue PCR. The positive bands has been marked by an arrow (D) EtBr stained gel showing completely digested DNA from WT and transgenic lines OEIF1 and OEIF2. 30 µg of genomic DNA from both WT rice and transgenic lines over-expressing OsIF was digested with NcoI and SpeI and transferred onto the nylon membrane; (E) Southern blot showing fallout in both the transgenic lines. Radiolabeled nested OsIF gene was used as a probe; (F) qRT-PCR analysis of wild-type (WT) and transgenic lines OEIF1, OEIF2 and KDIF1, KDIF2, showing increased and decreased accumulation of OsIF transcript in the over-expressed and knock-down transgenic lines than in the WT, respectively. Higher expression of OsIF was observed in OEIF2 in comparison to OEIF1. All data represent mean ± SD of three independent experiments. Statistically significant differences were determined using two-tailed paired Student's t-test as compared to control conditions and indicated by $*p < 0.05$.

Supplementary Figure S3:

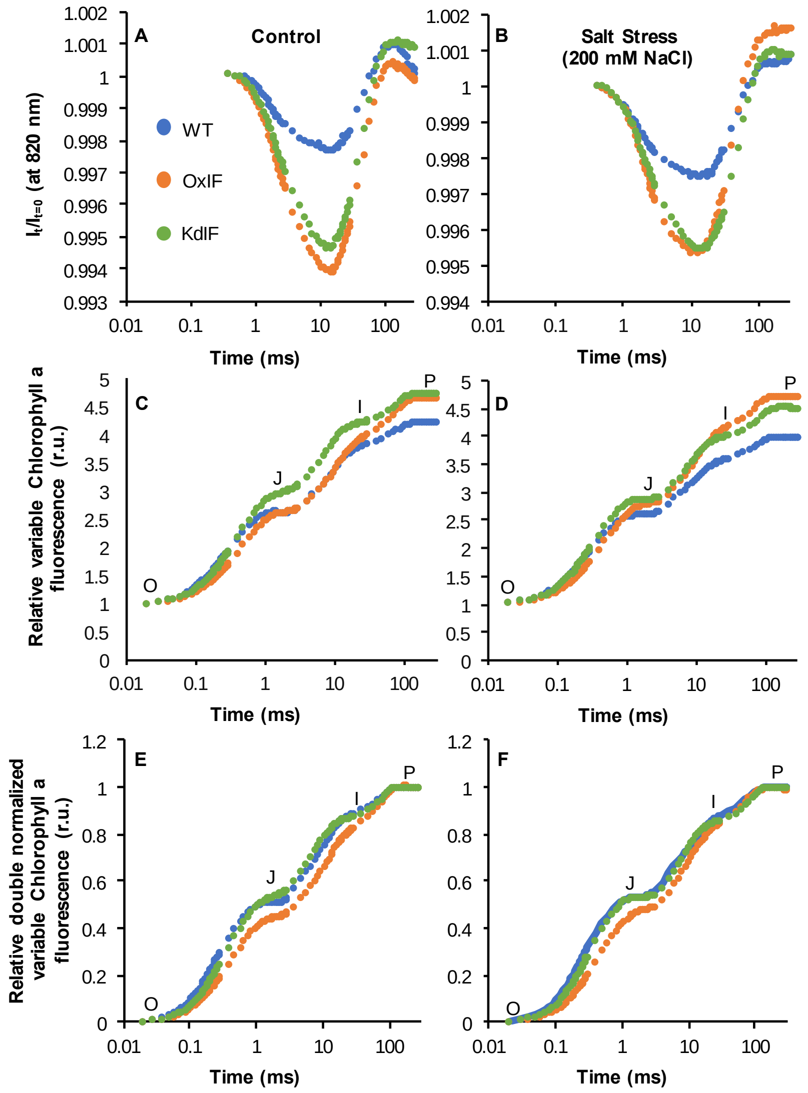

SupplementaryFigureS3: Relative 820 nm transmittance and Chlorophyll *a* fluorescence changes in WT, OEIF2 and KDIF2 plants under control and salt treatment. 820 nm transmittance curves of WT, OEIF2 and KDIF2 rice plants under (A) control and (B) salt stress. Simultaneously-measuredChlorophyll*a* fluorescencenormalized in (C) control and (D) salt stress condition; (E) Double normalized induction curves. Samples were dark adapted overnight before measurements. The curves are averages of six different samples.

Supplementary Figure S4:

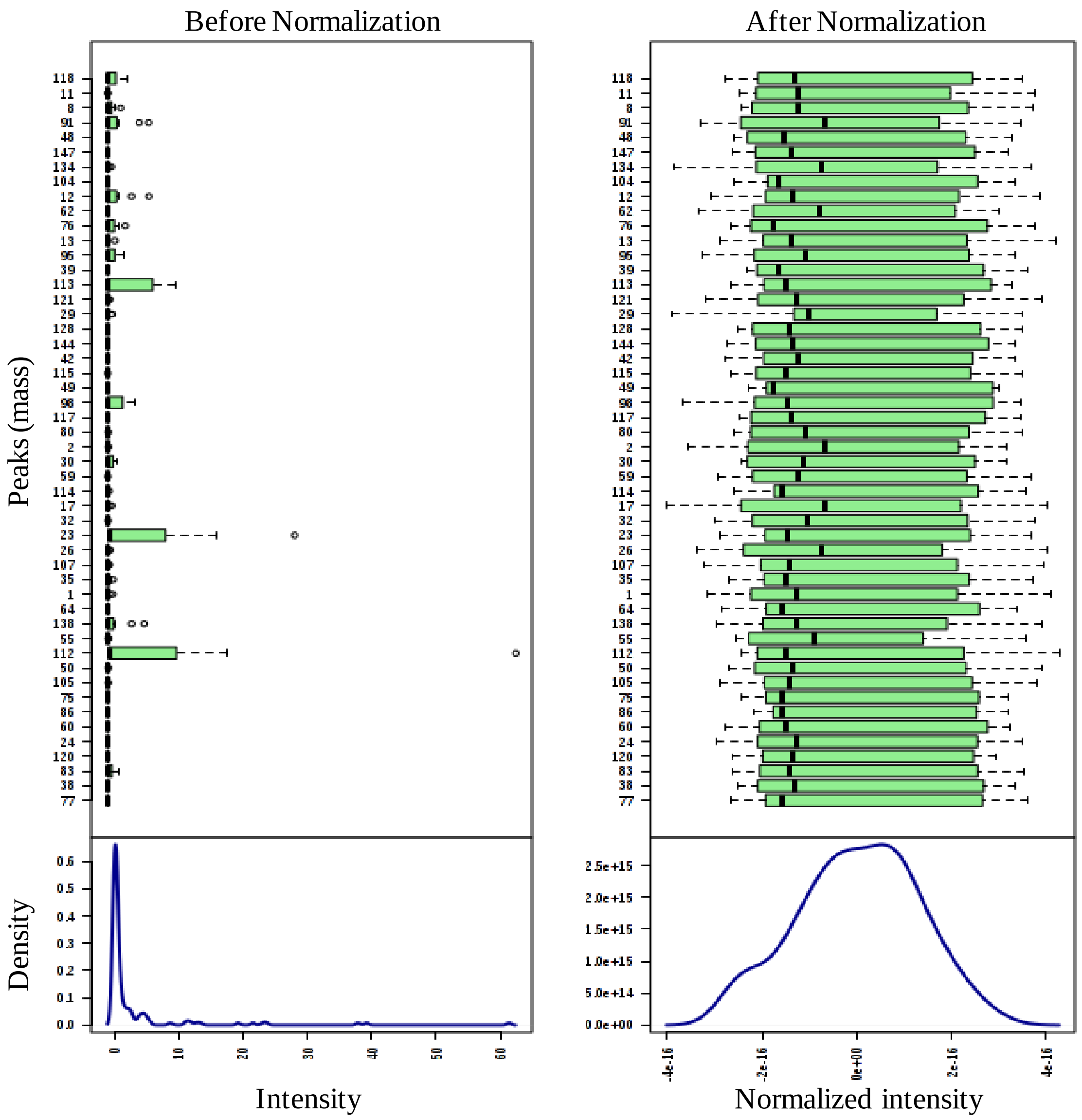

Supplementary Figure S4: Box plots and kernel density plots before and after normalization. The box plots show significant 50 features/sample while density plots are based on all the features. Normalization was carried out in three steps (i) Row-wise normalization: Normalization by a reference feature, (ii) Column-wise normalization: Log Normalization, (iii) Pareto scaling: Mean-centred and divided by the square root of standard deviation of each variable.

<u>Supplementary Figure S5</u>:

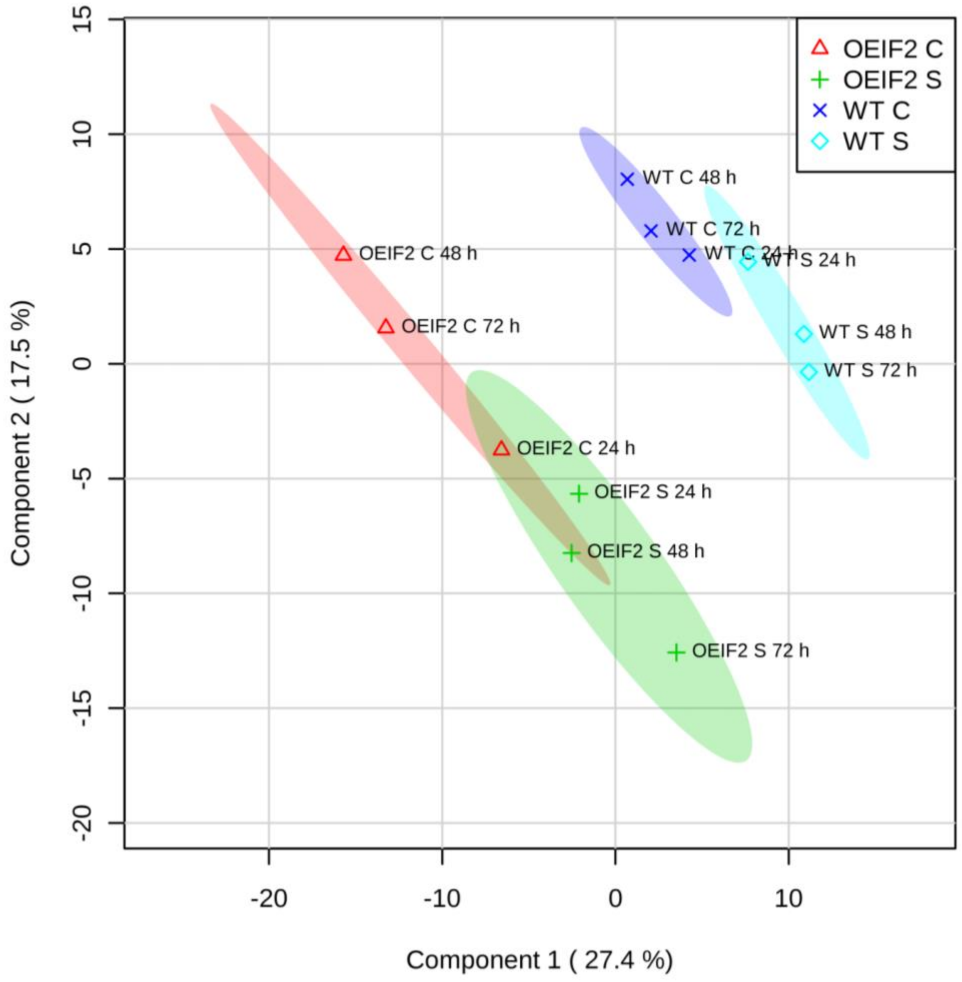

Supplementary Figure S5: Partial Least Squares Discriminant Analysis (PLS-DA):2D score plot of metabolome of 7 d old WT and OEIF2 rice seedlings grown under non-stress and under 200 mM NaCl for three different durations. Wild-type: under control (Dark blue) and stress (Light blue) conditions; and transgenic OEIF2: under control (Red) and stress (Green) conditions. Control and stressed samples are represented as C and S, respectively; duration of stress are 24 h, 48 h and 72 h that have been mentioned in the score plots. The variance is shown in bracket.

<u>Supplementary Figure S6</u>:

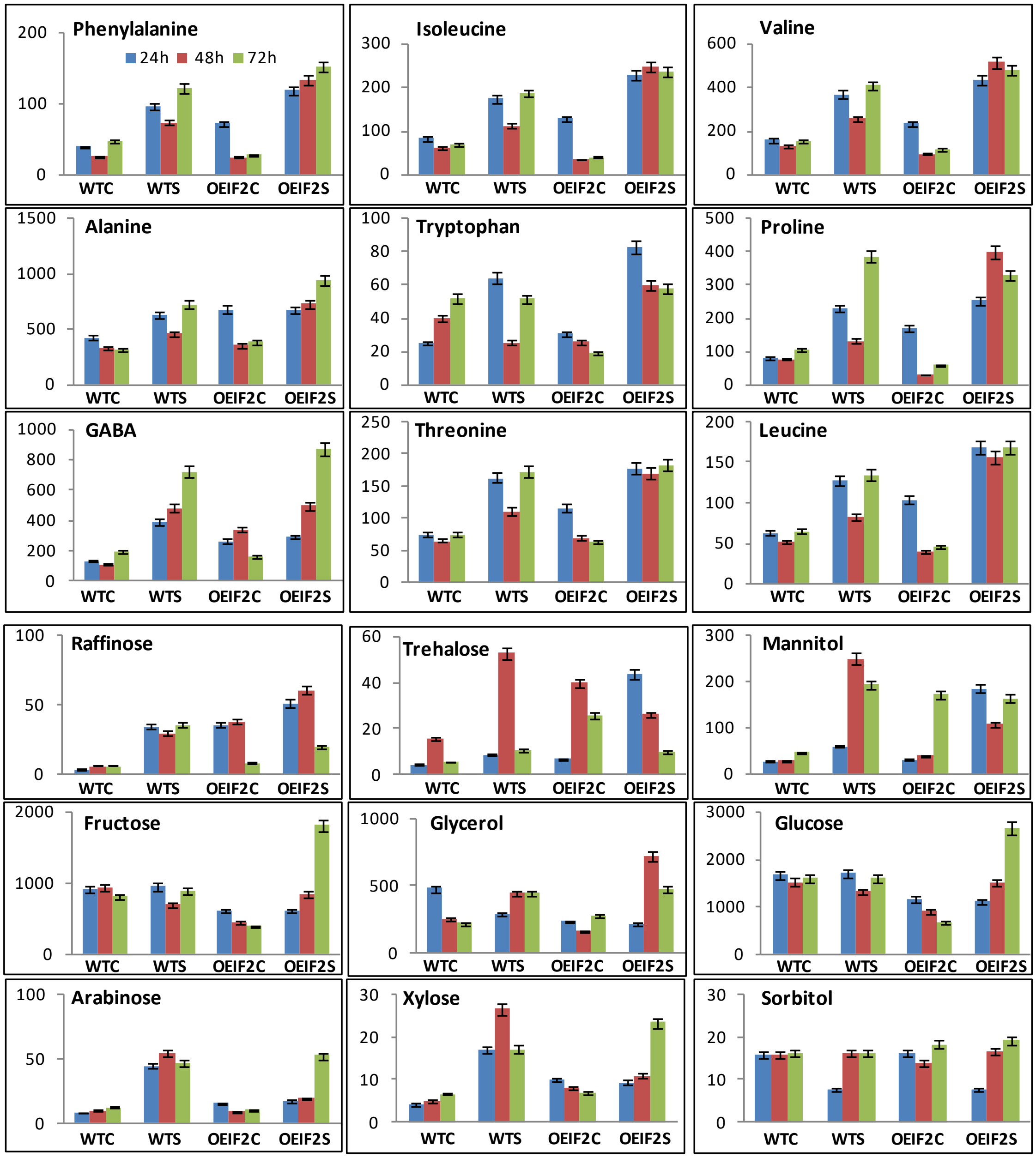

Supplementary Figure S6:Key amino acids and sugar metabolites of WT and IF transgenic plants identified by GC-MS which show major differences during stress with respect to the controls. Each data point represents the average of at least three biological replicates with the error bar representing the standard deviation. WTC: wild-type under control; WTS: wild-type under stress; OEIF2C: OEIF2 transgenic plant under control; OEIF2S: OEIF2 under stress.

Supplementary Table S1: $I_t/I_{t=0}$ **(at 820 nm) values for wild-type (WT), over-expression (OEIF2) and knock-down (KDIF2) plants under control and salt stress conditions.**

Time (ms)	WT Control	Time (ms)	WT Salt	Time (ms)	OEIF2 Control	Time (ms)	OEIF2 Salt	Time (ms)	KDIF2 Control	Time (ms)	KDIF2 Salt
**0.4**	1	0.4	1	0.4	1	0.4	1	0.4	1	0.4	1
**17**	0.99752±0.0004	14	0.99743±0.0005	15	0.99384±0.0007	13	0.99562±0.0005	13	0.99459±0.0005	14	0.99541±0.0004
**300**	0.99957±0.0015	300	1.00082±0.0009	300	0.99976±0.0013	300	1.00021±0.0029	300	1.00080±0.0009	300	1.00082±0.0008

Supplementary Table S2: Chlorophyll *a* fluorescence in wild-type (WT), over-expression (OEIF2) and knock-down (KDIF2) plants under control and salinity stress conditions.

	WT		OEIF2		KDIF2	
**Time (ms)**	**Control**	**Salinity**	**Control**	**Salinity**	**Control**	**Salinity**
**0.02 (O)**	1	1	1	1	1	1
**1 (J)**	2.58±0.1	2.52±0.13	2.45±0.03	2.55±0.03	2.79±0.07	2.76±0.05
**10 (I)**	3.34±0.14	3.18±0.17	3.33±0.05	3.53±0.06	3.87±0.01	3.58±0.09
**300 (P)**	4.20±0.25	3.94±0.14	4.64±0.06	4.68±0.13	4.71±0.06	4.48±0.13

Supplementary Table S3:Table showing relative concentration of 523 metabolites (159 known and 364 unknown) in triplicate. WT – Wild-type, OEIF2 –*OsIF* over-expressing transgenic line, C - Control, S – Stress: Attached as excel sheet.

Supplementary Table S4:Table showing primer sequences used in study. *qOsAct*: rice actin primers, *qOsIF*: rice intermediate filament primers, *OsIF*: rice intermediate filaments primers, restriction sites are shown in bold italicised fonts in the sequence.

Primer	Sequence 5'-3'
Primers for qRT-PCR	
qOsACT-F	CAGCCACACTGTCCCCATCTA
qOsACT-R	AGCAAGGTCGAGACGAAGGA
qOsIF-F	CTGTCGAAGCGATGAACTGA
qOsIF-R	TTGCGTCTGCCATAGACAAC
Primers for overexpression construct	
OsIFNcoI-F	CATG***CCATGG***CGCCGATTTCAGAGAGGGC
OsIFSpeI-R	G***ACTAGT***TCATCGCTTCGACAGCATAG
OsIFSpeI-R (without stop codon)	***GACTAGT***TCGCTTCGACAGCATAG
Primers for knockdown construct	
OsIFAscI-F	***GGCGCGCC***AAGGATGAGCAGCTGCG
OsIFSwaI-R	***ATTTAAAT***AAGAATTGATCTTCTCATGCC
OsIFSpeI-F	GG***ACTAGT***AAGGATGAGCAGCTGCG
OsIFBamHI-R	CG***GGATCC***AAGAATTGATCTTCTCATGCC
